# 304. Immunoproteasome Inhibition Augments Endoplasmic Reticulum Stress and Enhances Disease During Coronavirus Respiratory Infection

**DOI:** 10.1093/ofid/ofad500.376

**Published:** 2023-11-27

**Authors:** Jacob C Steigmann, Lauren Suttenberg, Xiaofeng Zhou, Jason Weinberg

**Affiliations:** University of Michigan, Ann Arbor, Michigan; University of Michigan, Ann Arbor, Michigan; University of Michigan, Ann Arbor, Michigan; University of Michigan, Ann Arbor, Michigan

## Abstract

**Background:**

Inflammation contributes to disease in patients with SARS-CoV-2 infection. The immunoproteasome (IP), an inducible component of the ubiquitin-proteasome system, contributes to the diversity of T cell epitopes generated during infection and production of antiviral and pro-inflammatory cytokines. Like the standard proteasome, the IP degrades damaged and misfolded proteins to minimize proteotoxic cell damage, which may occur via endoplasmic reticulum (ER) stress pathways. IP subunit expression increases in lungs of patients with SARS-CoV-2 infection. We previously demonstrated increased IP activity in mice infected with murine hepatitis strain 1 (MHV-1), a murine coronavirus. However, specific contributions of the IP to coronavirus pathogenesis are not yet defined. We used MHV-1 to test the hypothesis that increased IP activity is a protective host factor during acute coronavirus infection.

**Methods:**

C3H.HeJ mice infected with MHV-1 were treated with vehicle or ONX-0914 (ONX), an IP inhibitor. We monitored weight loss and survival following infection and harvested organs at multiple times. Bone marrow-derived dendritic cells (BMDC) from C3H.HeJ mice were infected with MHV-1 in the presence of ONX. We used qPCR to assay viral load and expression of cytokines (IFN-β, IFN-γ, TNF-α, IL-6, IL-1β) and genes involved in ER stress pathways (CHOP, BiP, Grp94, Calreticulin, GADD34).

**Results:**

IP inhibition enhanced weight loss and mortality following MHV-1 infection but did not substantially affect viral replication. Virus-induced expression of TNF-α and IL-1β was lower in the lungs of ONX-treated mice, while TNF-α expression was higher in livers. Expression of ER stress pathway genes was significantly greater in both lungs and livers of infected, ONX-treated mice compared to controls early in the course of infection. IP inhibition did not affect viral replication in BMDC, but it did enhance expression of IFN-β, TNF-α, and GADD34.

**Conclusion:**

IP inhibition enhanced MHV-1-induced disease but did not affect viral replication. IP inhibition had modest effects on virus-induced inflammation. Increased evidence of ER stress in mice and cells treated with ONX suggests that the IP minimizes harmful proteotoxic stress, particularly in the liver, during acute coronavirus infection.

**Disclosures:**

**All Authors**: No reported disclosures

